# A Torsion-Bending Antagonistic Bistable Actuator Enables Untethered Crawling and Swimming of Miniature Robots

**DOI:** 10.34133/research.0116

**Published:** 2023-04-11

**Authors:** Nan Hu, Bo Li, Ruiyu Bai, Kai Xie, Guimin Chen

**Affiliations:** ^1^ State Key Laboratory for Manufacturing Systems Engineering and Shaanxi Key Laboratory of Intelligent Robots, School of Mechanical Engineering, Xi’an Jiaotong University, Xi’an 710049, China.; ^2^ School of Aerospace Science and Technology, Xidian University, Xi’an 710126, China.

## Abstract

Miniature robots show great potential in exploring narrow and confined spaces to perform various tasks, but many applications are limited by the dependence of these robots on electrical or pneumatic tethers to power supplies outboard. Developing an onboard actuator that is small in size and powerful enough to carry all the components onboard is a major challenge to eliminate the need for a tether. Bistability can trigger a dramatic energy release during switching between the 2 stable states, thus providing a promising way to overcome the intrinsic limitation of insufficient power of small actuators. In this work, the antagonistic action between torsional deflection and bending deflection in a lamina emergent torsional joint is utilized to achieve bistability, yielding a buckling-free bistable design. The unique configuration of this bistable design enables integrating of a single bending electroactive artificial muscle in the structure to form a compact, self-switching bistable actuator. A low-voltage ionic polymer–metal composites artificial muscle is employed, yielding a bistable actuator capable of generating an instantaneous angular velocity exceeding 300 °/s by a 3.75-V voltage. Two untethered robotic demonstrations using the bistable actuator are presented, including a crawling robot (gross weight of 2.7 g, including actuator, battery, and on-board circuit) that can generate a maximum instantaneous velocity of 40 mm/s and a swimming robot equipped with a pair of origami-inspired paddles that swims breaststroke. The low-voltage bistable actuator shows potential for achieving autonomous motion of various fully untethered miniature robots.

## Introduction

Miniature robots have the unique capability to access and navigate in difficult-to-reach, confined spaces to perform various tasks [1,2]. Due to the limited onboard space, most of these robots require electrical or pneumatic tethers to connect to power supplies outboard [3,4], which considerably limits their maneuverability. To increase load capacity of pneumatic and electrical actuators for carrying heavy components such as batteries, microprocessors and actuators themselves can eliminate the need for tethers but always leads to substantially scaling up the actuators as well as the robots [5]. Due to its high energy density, combustion-powered actuation was successfully employed in soft robots to realize untethered jumping and autonomous operation [6,7]. Although it shows strong potential for further applications in untethered robots, further progress is required to achieve more controllable combustion with a miniature structure. There have also been a number of studies utilizing external physical fields or directed energy beams to power miniature robots so as to place bulky components outboard without physical connections [8–12], for example, the 3D-printed ferromagnetic robot navigating in an alternating magnetic field [13,14], the origami robot changing its configuration at an external thermal stimulus [15,16], the terrestrial robot crawling and jumping upon laser-induced local thermal actuation [17], and the soft robot rolling in a constant-humidity environment [18], to name a few. Although higher maneuverability can be obtained by eliminating tethered connections, extra devices are required to generate the tunable fields, leading to very large robotic systems. Developing an onboard actuator that is small in size and powerful enough to carry all the components onboard is still a major challenge for achieving autonomous motion of fully untethered miniature robots [19].

Bistable mechanisms can trigger a dramatic energy release during switching between the 2 stable states [20,21] and thus have been employed in actuator designs to increase the instantaneous power density [22], for example, the cheetah-like galloping crawlers [23], the pneumatic bistable soft actuator for autonomous control of crawling [24], the bistable actuator capable of bistable-continuous motion [25], the jumping robot [26,27], and the self-propelling swimming robot [28]. Generally speaking, 2 independent active elements or chambers are required to switch the bistable mechanisms back and forth, respectively, leading to complicated control designs. Besides, most bistable designs utilize buckling behaviors of flexible beams [29] or membranes [30–33], which is not favored because the existence of multiple buckling modes may induce unpredictable behaviors [34–36].

In this work, the antagonistic action between torsional deflection and bending deflection in a lamina emergent torsional (LET) joint is explored, from which a buckling-free bistable design is obtained. LET joints have been extensively adopted as surrogate joints in developable mechanisms [37,38] and origami-inspired designs [39,40] because they can be easily fabricated from planar materials but provide out-of-plane motion [41]. In a LET joint, the compliant beams are intended to undergo torsional deflection for producing out-of-plane motion [41], but they are also vulnerable to in-plane bending, which is known as parasitic motion. While many researchers seek for solutions to minimize or even eliminate this parasitic motion, the current work harnesses it for creating a strain energy barrier so as to achieve a bistable behavior. The unique configuration of this bistable design enables implanting of a single bending electroactive artificial muscle in the structure to form a compact, self-switching bistable actuator.

Ionic polymer–metal composite (IPMC) is chosen in this work because it can be driven at low voltage, which eliminates the need for bulky high-voltage electronic devices and thus is beneficial to developing miniature robot. However, the slow response of IMPC could not provide effective thrust for a robot to overcome the friction resistance for crawling, or the fluid resistance for swimming. Therefore, a torsion-bending antagonistic bistable mechanism is proposed to work with IPMC for actuation of miniature robots. The bistable mechanism can trigger a dramatic energy release during switching between the 2 stable states, which can substantially increase the instantaneous power density of IPMC for overcoming the resistance. Although IPMC often requires aqueous environments, the new packaging technology [42] enables IPMC to be operated well in dry environments.

We demonstrated the utility of the bistable actuator in 2 robots: (a) An untethered crawling robot, whose gross weight is 2.7 g including the integrated low-voltage battery and control circuit. The robot advances by utilizing the directional friction effects of its feet. (b) An untethered swimming robot, which employs a pair of origami-inspired paddles to amplify the stroke of the bistable actuator. The bistable actuator is biased to facilitate directional propelling of the robot showing a breaststroke swimming mode.

## Results

### Bistable design

**Fig. 1.**
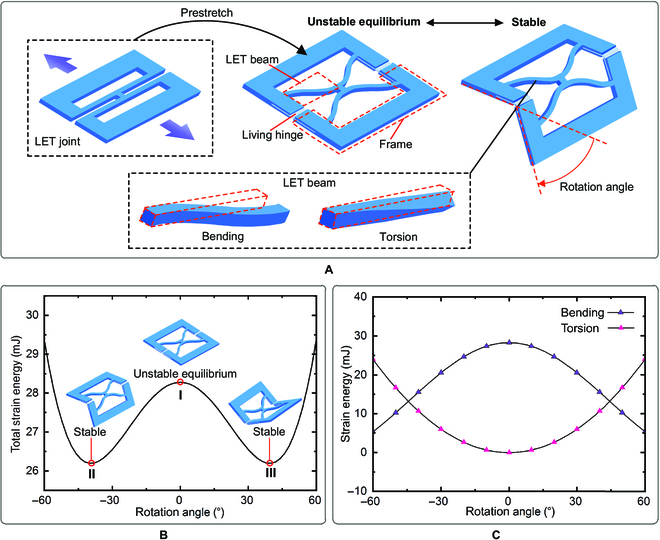
Torsion-bending antagonistic bistable design. (A) Schematic of torsion-bending antagonistic bistable design. It is built by attaching 2 living hinges in between the frames of a prestretched LET joint. The prestretch yields bending deflection in the 4 LET beams, while the rotation of the LET joint produces torsional deflection. (B) A strain energy barrier. The maximum position (I) is produced through prestretch, and the strain energy drops when the joint rotates in either direction, generating 2 local minimum positions (II and III). The 2 local minimum positions on the total strain energy curve correspond to 2 stable states while the maximum position to unstable equilibrium state of the design. (C) Strain energy curves of the LET beams due to bending and torsion.

As sketched in Fig. 1A, the bistable design is composed of a LET joint and 2 living hinges. The living hinges are attached between 2 frames after the LET joint being prestretched. This prestretch yields in-plane bending of 4 LET beams and produces a strain energy barrier (corresponding to an unstable equilibrium state) that is critical for achieving bistability, as illustrated in Fig. 1B. The total strain energy *E*_Tol_ stored in the LET beams is

$$E_{\mathrm{Tol}}=E_{\mathrm{LB}}+E_{\mathrm{LT}}$$ (1)

where *E*_LB_ is the strain energy due to bending and

$$E_{\mathrm{LB}}=\int_{0}^{Y_{x}}F_{L}{\mathrm{dY}}_{L}$$ (2)

in which *F*_L_ and *Y*_L_ are the force and displacement produced by prestretch of the LET joint, and *E*_LT_ is the strain energy due to torsion

$$E_{\mathrm{LT}}=\int_{0}^{\theta_{x}}\mathrm{Md\theta}$$ (3)

in which *M* and *θ* are the moment and rotation angle of the LET joint. An approximately linear relation between *M* and *θ* was observed, which was captured by a linear torsion model [43]. The highly nonlinear relation between *F*_L_ and *Y*_L_, as discussed in [39], was calculated by the chained beam constraint model [44]. The detailed static model is provided in Note S1.

When the LET joint rotates and deviates from this unstable state, the LET beams are twisted and the strain energy due to torsion increases, while the strain energy due to bending decreases. If the decrease of the strain energy due to bending dominates the change of the total strain energy (Fig. 1C), the joint exhibits a bistable behavior, as shown in Fig. 1B. The antagonistic action between torsion and bending provides us a new way to achieve bistability without buckling.

### Self-switching bistable design and characterization

**Fig. 2.**
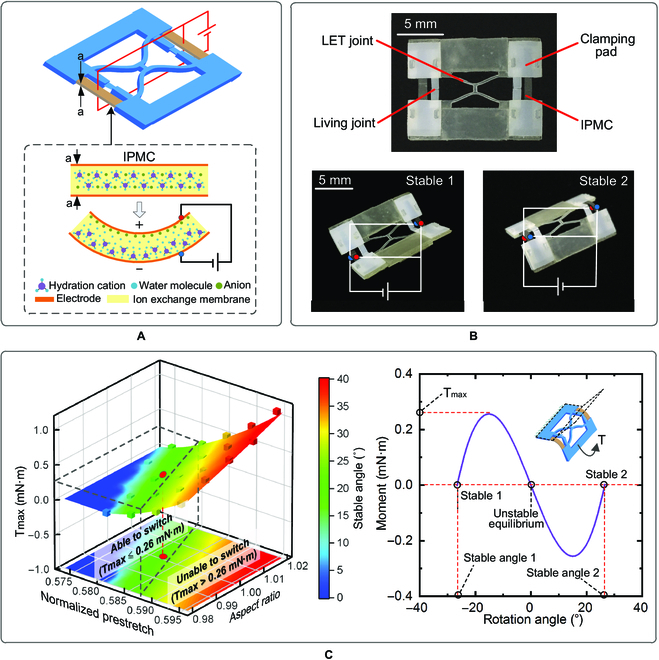
Design for self-switching. (A) Principle of self-switching. The living hinges are replaced by 2 low-voltage electroactive IPMC strips to provide bending actuation. (B) Prototype of the bistable actuator. IPMCs are attached to the frames by clamping pads. The switching between the 2 stable states is achieved by alternating the voltage applied on IPMCs. (C) Parameter determination of the bistable LET design. Left: The switching moment and stable angle are plotted with respect to the aspect ratio of beam cross-section and the normalized prestretch deflection, in which the zone surrounded by the dashed lines represents the feasible region (*T*_max_ ≤ 0.26 mN·m). The experimental results are represented by small cubes, and the optimal design is marked as a red dot. Right: The moment curve of the finalized bistable design.

IPMC is a kind of electroactive polymer that can provide large bending deflection when subject to a low-voltage stimuli (usually 3 to 5 V) (the working principle and the preparation process of IPMC are provided in Note S2) [45]. The IPMC artificial muscle is composed of 2 narrow IPMC strips (Fig. 2A) that are symmetrically arranged in the bistable LET joint and electronically connected to the same control circuit (Fig. 2B). The 2 IPMC strips bend synchronously, each of which can generate a minimum actuation moment of 0.13 mN·m during its lifetime (see the experimental setup and the test results in Note S2). This moment is used to determine the cross-section of the LET beams and the prestretch deflection to maximize the switching moment *T*_max_ and also to guarantee self-switching. Figure 2C plots the switching moment with respect to the aspect ratio of beam cross-section and the normalized prestretching deflection, from which the length, prestretching, width, and thickness of 4.6, 2.68, 0.5, and 0.5 mm, respectively, are determined (red dot in the left of Fig. 2C; the geometrical and material parameters are listed in Table S3); the bistable behavior is plotted on the right of Fig. 2C. The experimental results for the switching moment and the stable angle of different bistable designs are overlaid on the predicted ones in Fig. 2C for the purpose of comparison. The experimental results agree well with the predicted ones, with the relative errors for the stable angles and the maximum moments less than 5% and 9%, respectively.

**Fig. 3.**
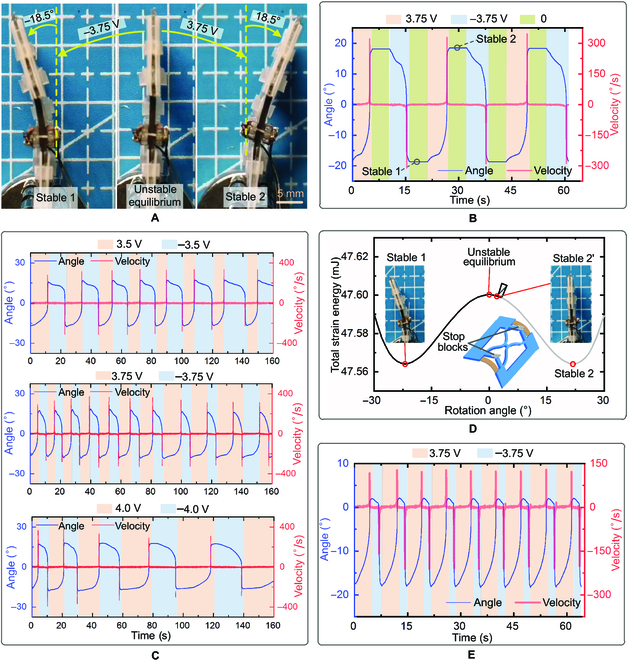
Characterization of self-switching. (A) Switching of bistable actuator between the stable states and the unstable equilibrium state. The bottom end of bistable actuator is fixed and the deflection of the free end is recorded. (B) Rotation angle and angular velocity of bistable actuator under an alternative voltage. The sign of the applied voltage is distinguished by the background color. (C) Rotation angle and angular velocity of bistable actuator with voltages of different amplitude (3.5, 3.75, and 4 V). (D) Total strain energy curve of the bistable actuator with stop blocks. Stable 2' (the rotation angle is 2°) is the new stable state between the unstable equilibrium stable and stable 2. (E) Rotation angle and angular velocity of the bistable actuator with stop blocks.

In characterizing the bistable actuation, the bistable actuator is fixed on one end and left free on the other end. IPMCs are powered by a square-wave voltage (the actuation period is determined by a circuit illustrated in Fig. S8A), and the deflection at the free end is recorded. Figure 3A demonstrates the 3 states of the bistable actuator switching between stable 1 and stable 2. Figure 3B plots the rotation angle and the angular velocity of the bistable actuator with respect to time. It shows that the actuator can be switched from stable 1 to stable 2 by applying a 3.75-V voltage and switched back by applying a −3.75-V voltage, and the measured rotation angle from the unstable equilibrium state to 2 stable states is 18.5°. During the bistable actuator rotates from stable 1 to the unstable equilibrium state, the work done by IPMCs mainly converts to strain energy in LET beams, thus a small angular velocity is achieved. Once the bistable actuator surmounts the unstable equilibrium state, the stored strain energy starts to release, causing a sudden jump to stable 2 with an instantaneous angular velocity exceeding 300 °/s. The bistable actuator remains the stable state even the voltage is off (Movie S1). A similar process is observed when switching the bistable actuator from stable 2 to stable 1.

The energy density *W* is calculated as

$$W=\frac{\int_{\theta_{1}}^{\theta_{2}}T_{a}\mathrm{d\theta}}{m_{a}}$$ (4)

where *T*_a_ is the measured moment of the bistable actuator (the experimental setup and the measured results are shown in Fig. S7), and *m*_a_ is the mass (0.2 g). From one stable state (*θ*_1_) to another (*θ*_2_), the energy density is 0.5 mJ/g. It takes *t* = 10 s for the actuator to be switched between the 2 stable states, and the average power density *P*_M_ (the energy density divided by time) can be calculated as

$$P_{M}=\frac{W}{t}=0.05\;\mathrm{mW}\;/\;g$$ (5)

The instantaneous power density *P*_I_ is determined as

$$P_{I}=\frac{T_{a}V_{a}}{m_{a}}$$ (6)

where *V*_a_ is the measured instantaneous velocity (rad/s) of the bistable actuator. The maximum of *P*_I_ reaches 14 mW/g, which is 280 times as much as *P*_M_. This feature is achieved by the dramatic energy release of the bistable mechanism.

In general, increasing the amplitude of the applied voltage on IPMC reduces the duration of action required to switch the bistable actuator but deteriorates the bending capability of IPMC resulted from the loss of solvent (water). To determine a suitable amplitude, the characteristics of the bistable actuator under different voltages are tested. Figure 3C displays the switching cycles of the bistable actuator in 160 s when subject to a voltage input of 3.5, 3.75, and 4 V, respectively (Movie S2). The average period for 3.5 V is 1.33 times longer than that for 3.75 V in 150 s. Although the period of the first cycle for 4.0 V is a little shorter than that for 3.75 V, the average period is 1.81 times than that of 3.75 V in 160 s, which is attributed the rapid loss of solvent in IPMC when subject to a high voltage. The experimental results of the lifetime test for the bistable actuator under different voltages are shown in Fig. S7C. It shows that the lifetime of the actuator under voltages of 3.5, 3.75, and 4.0 V are 17, 15, and 7 min, and the average actuation period during the corresponding lifetime are 40 s, 29 s and 47 s, respectively. The experimental results for the actuation moment of bistable actuator with different voltage and normalized prestretch are plotted in Fig. S7D and E. Given an overall consideration, 3.75 V is selected as the voltage input for bistable actuator in the following.

The 2 stable states of the bistable actuator shown in Fig. 3A are symmetric with respect to the unstable equilibrium state, providing similar actuations for forward switching and backward switching. For directional propelling, a biased bistable behavior is often favored. By placing stop blocks to prevent the actuator from switching to stable 2, a new stable position in between the unstable equilibrium stable and stable 2 (e.g., stable 2' at the position with a rotation angle of 2° shown in Fig. 3D) is created. This biased bistable actuator undergoes an energy storage-dominated process from stable 1 to stable 2' while an energy release-dominated process from stable 2' to stable 1, producing an intentionally asymmetric actuation for forward switching and backward switching. Figure 3E records the actuation properties of this biased bistable actuator when applying a square-wave voltage. It shows that the voltage duration from stable 2' to stable 1 (−3.75 V) is considerably shorter than that from stable 1 to stable 2' (3.75 V), and the maximum angular velocity is nearly 5 times of that from stable 1 to stable 2' (Movie S3).

### Untethered crawling

**Fig. 4.**
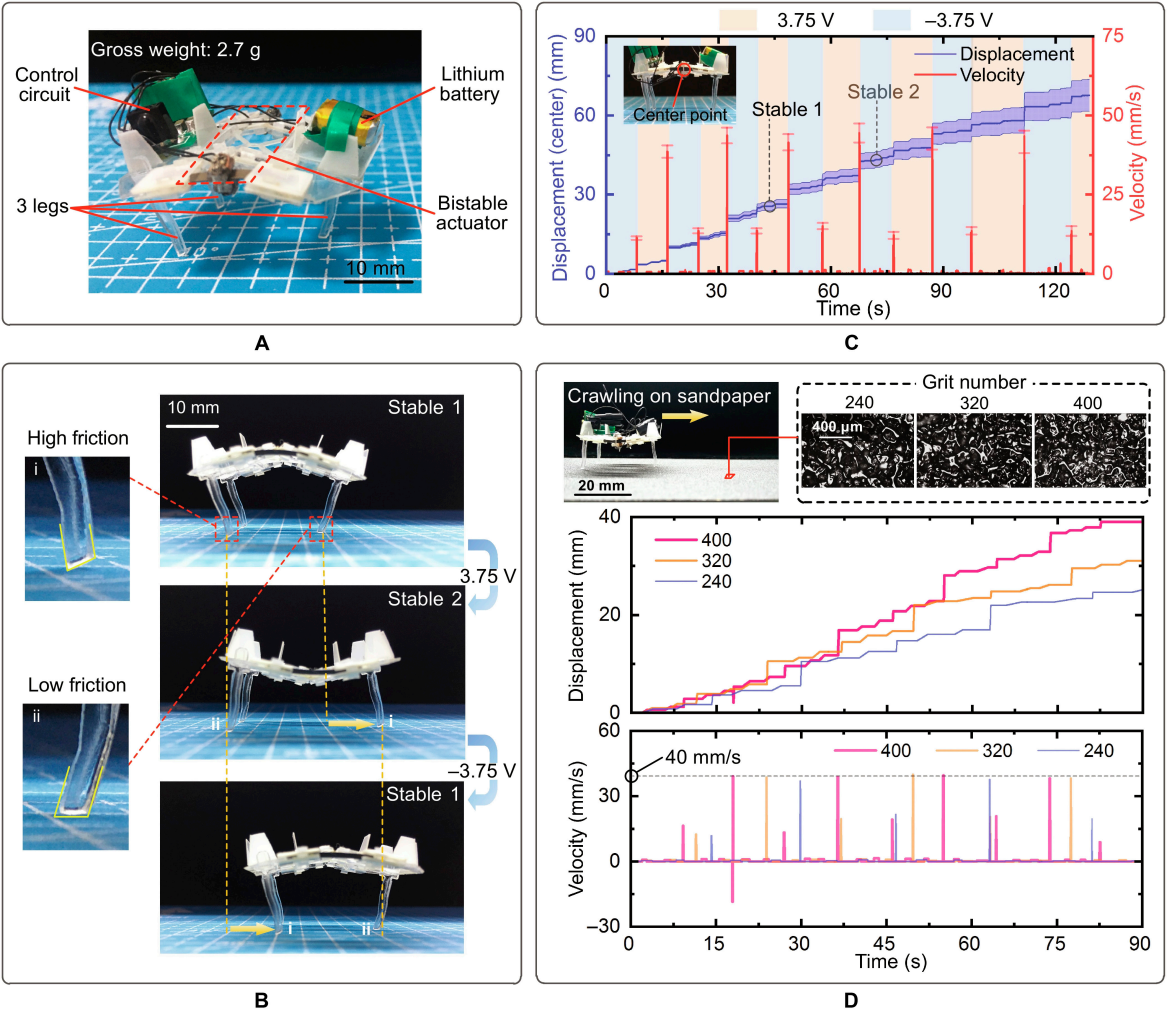
Bistable crawling robot. (A) Prototype of the bistable crawling robot. An untethered 3-leg robot employing the bistable actuator, with a carry-on lithium battery and a carry-on control circuit is fabricated. (B) Directional locomotion of robot: from stable 1 to stable 2, the bistable actuator switches downward (3.75 V), during which the rear feet are pinned to ground due to the sharp corner while the front foot moves forward; from stable 2 to stable 1, the bistable actuator switches upward (−3.75 V), during which the front foot is pinned to ground while the rear feet move forward. (C) Measured displacement and velocity of the center point of the robot. The sign of the applied voltage is distinguished by the background color. (D) Crawling on sandpaper with different grit numbers (240, 320, and 400).

A 3-leg crawling robot is developed utilizing the bistable actuator (Fig. 4A). A low-voltage lithium battery and a control circuit are carried on the robot, making it an all-in-one untethered robot whose overall mass is 2.7 g (Table S4). The torso dimensions of the robot are 28 mm (length) × 23 mm (width) × 22 mm (height). Each leg is laser cut from an acrylic sheet to form a sharp corner at the heel of the foot. When the back of the robot is up, the rear feet are pinned to the ground with their sharp corners due to the high friction in forward direction, while the front foot contacts the ground with the flat part leads to low friction, thus the front leg slides forward and the rear legs are anchored during switching down. Similarly, as the back of the crawler is down, the front foot is pinned to the ground with its sharp corner while the rear feet contact the ground with their flat parts, so that the front foot is anchored and the rear feet move forward.

By alternating the voltage applied on IPMCs (the control circuit is described in Note S4), the bistable actuator is switched back and forth and the robot moves forward by alternatively extending its front foot and rear feet. Figure 4B demonstrates different states of the robot in a full crawling cycle. The measured displacement and velocity of the robot crawling on a flat ground are plotted in Fig. 4C (Movie S4). One can find that the robot produces a maximum instantaneous velocity of 40 mm/s during switching to stable 2 and no backwards movement is observed. In 1 crawling cycle, the average voltage duration of the robot is 18.6 s and the average displacement is 10.6 mm.

To demonstrate the ability to crawl on different grounds, the crawling robot was tested on sandpaper with different grit numbers (the larger the grit number is, the smoother the surface is), the measured displacements and velocities are plotted in Fig. 4D (Movie S5). The average velocities of the robot crawling on the sandpaper with grit number of 240, 320, and 400 are 0.27, 0.34, and 0.43 mm/s, with the maximum instantaneous velocities are 39.95, 40.05, and 40.11 mm/s, respectively. The average crawling velocity of the robot decreases with the increase of the ground roughness, while the maximum instantaneous velocity remains almost unchanged.

### Untethered swimming

**Fig. 5.**
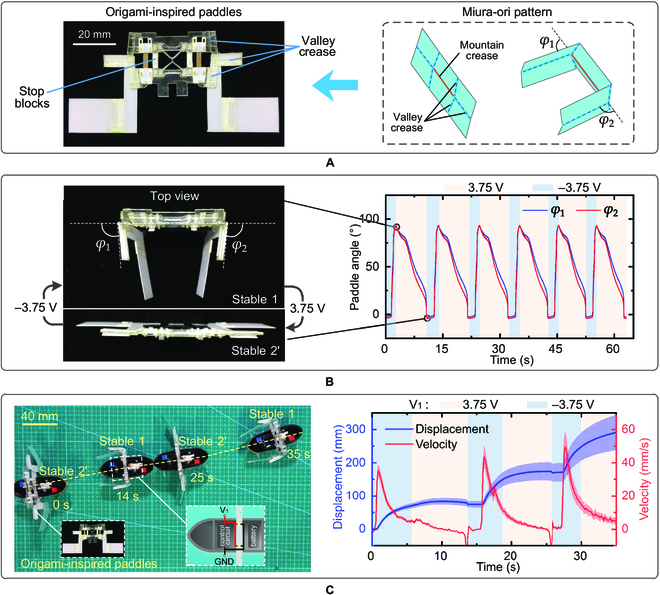
Bistable swimming robot. (A) Origami-inspired paddles. The Miura-ori pattern is selected to amplify the rotation angle of the bistable actuator and the mountain crease (red solid line) is actuated by the bistable actuator. (B) Measured paddle angles *φ*_1_ and *φ*_2_. The stop blocks are employed to confine a workspace as the bistable actuator switching between stable 1 and stable 2' (the nearly flat state). The sign of the applied voltage is distinguished by the background color. (C) Left: Propelling of the robot by the origami-inspired paddles. The robot can swim the breaststroke in a straight line under an alternative voltage. Right: Recorded propelling displacement and velocity.

A self-propelling robot is developed utilizing the bistable actuator equipped with a pair of origami-inspired paddles. The Miura-ori pattern [39,46,47] is selected for the paddles, which amplifies the rotation angle of the bistable actuator from 20.5° (the rotation angle of the mountain crease marked by a solid red line in Fig. 5A) to 94° (paddle angles *φ*_1_ and *φ*_2_, see the design process in Note S5). The mountain creases of the 2 paddles are collinear and synchronously actuated by the bistable actuator. Two stop blocks are employed to prevent the mountain crease from being folded as a valley crease [39] so that the bistable actuator only works between stable 1 to stable 2' (the nearly flat state). The measured paddle angles *φ*_1_ and *φ*_2_ are changed in between −2° and 92° when actuated by the bistable actuator, as plotted in Fig. 5B (Movie S6). The required voltage duration from stable 2' to stable 1 (deploying) is one-fifth of that from stable 1 to stable 2' (folding) on average.

The propelling of the swimming robot is illustrated in Fig. 5C (left). The bistable actuator first folds the paddles (from stable 2' to stable 1), during which the strain energy stored in the LET beams is suddenly released and generates a high propulsive force as the paddles propel backward and grab the water like swimming the breaststroke. Next, the actuator switches back to store strain energy (from stable 1 to stable 2'), during which the paddles are slowly deployed to minimize retracting the robot. Figure 5C (left) shows the swimming trajectory during a straight-line motion (Movie S7), and the average propulsion velocity is 0.15 body lengths per second (BL/s). Figure 5C (right) plots the displacement and velocity of the swimming robot in 3 swimming cycles, showing a maximum instantaneous velocity of 50 mm/s. The average propulsion distance in 1 cycle is 110 mm, while the average retracting distance is 9 mm. When the robot was loaded with 6 g of water, the average propulsion velocity reduced to 0.1 BL/s (Movie S7).

## Discussion

In this work, a buckling-free bistable mechanism was designed through utilizing the antagonistic action between torsional deflection and bending deflection in a LET joint. A low-voltage IPMC artificial muscle was implanted in the bistable mechanism, yielding a self-switching bistable actuator capable of generating an instantaneous angular velocity exceeding 300 °/s by a 3.75-V voltage and the instantaneous power density during switching between the 2 stable states is increased by 280 times of average power density. Two untethered robotic demonstrations using the bistable actuator are presented, including a 2.7-g weight crawling robot that can generate a maximum instantaneous velocity of 40 mm/s and a swimming robot equipped with a pair of origami-inspired paddles that swims 110 mm in one propulsion.

Table summaries different bistable actuators designed for robots. It can be seen that most of them are either tethered or relying on external physical fields. Besides the robots developed in this work, only the pneumatic bistable swimming robot (the largest one in the table) in [48] features untethered and self-powered by an onboard CO_2_ canister. However, the pneumatic components make it challenging to be miniaturized. So far, the robots utilizing the proposed bistable actuator are the smallest robots that are both untethered and self-powered.

**Table.** Comparison of bistable actuators for robots (sorted by largest dimension).

Largest dimension (mm)	Type of locomotion	Actuation	Tethered (T)/ untethered (U)	External physical fields (Y/N)	Reference
260	Swimming	Pneumatic	U	N	[48]
130 100 70	Swimming Grasping Crawling	Pneumatic	T	N	[23]
100	Crawling	Pneumatic	T	N	[24]
85	Crawling	Motor	T	N	[4]
70	Swimming	Shape memory polymer	U	Y	[28]
52 50	Jumping Grasping	Twisted-and-coiled actuator	T	N	[49]
45 28	Swimming Crawling	IPMC	U	N	This work
20	Crawling	Magnetic plate	U	Y	[50]
2	Jumping	Liquid crystal polymer	U	Y	[51]

IPMC may suffer from solvent loss, which considerably deteriorates the performance of the proposed bistable actuator. Other smart materials of low driving voltage, for example, shape memory alloys, shape memory polymers, and twisted and coiled polymer fibers, can be explored to be integrated with the bistable design to achieve more robust bistable actuators. In addition, multiple bistable actuators can be combined in parallel to form a multistable actuator that could be employed in an untethered miniature robot to fulfill complex tasks.

## Materials and Methods

### Materials

The preparation of IPMC is summarized in Note S2. The LET joints were laser-cut (CMA-6040, GDHAN’S YUEMING LASER GROUP) from polyurethane sheets whose material parameters are listed in Table S3. The clamping pads and the legs of the bistable crawling robot were laser-cut from polyethylene terephthalate plates (0.5 mm thick) and acrylic plates (0.8 mm thick), respectively. The cabin of the bistable swimming robot was 3D-printed using carbon fiber, and other parts were laser-cut from polyethylene terephthalate plates (0.3 mm thick). The living hinges were glued to the prestretched LET joint by organic adhesive (401, GUBAILI).

### Methods for characterizing actuators and robots

The deflections of IPMC and bistable actuator were recorded by a laser displacement sensor (IL-065, Keyence) and the actuation forces (moments) were recorded by a force sensor (GSO-10, Transducer Techniques). The experimental setup is shown in Figs. S6C and S7A and B. The motions of the crawling robot and the swimming robot were captured by a high-speed camera (DSC-RX100M3, SONY), and their trajectories were extracted from the screenshots of the videos.

### Batteries for bistable robots

For the crawling robot, a rechargeable lithium battery (501015, CHIPTECH) with a capacity of 80 mAh was employed, which can power the robot for about 6 min when fully charged. While for the swimming robot, a rechargeable lithium battery (401030, CHIPTECH) with capacity of 150 mAh was utilized, which can power the robot for about 10 min when fully charged.

## Data Availability

Data supporting the findings of this study are available in the main text or the Supplementary Materials.

## Supplementary Materials

Supplementary 1 Note S1. Fabrication and modeling of bistable mechanism. Note S2. Preparation and characterization of IPMC. Note S3. Characterization of bistable actuator. Note S4. Control of bistable actuator. Note S5. Design of origami-inspired paddles. Figure S1. Fabrication process of bistable structure. Figure S2. Torsion and bending of LET beams. Figure S3. Mechanical model of torsion-bending antagonistic bistable mechanism. Figure S4. Stable angles of bistable mechanisms with different geometrical and materials parameters. Figure S5. Experimental setups for testing the stable angle (a) and the moment (b) of the bistable mechanism. Figure S6. Preparation and characterization of IPMC. Figure S7. Characterization of bistable actuator. Figure S8. Control of bistable actuator. Figure S9. Miura-ori structure. Table S1. Theoretical and experimental results of stable angle and maximum moment of bistable designs plotted in Fig. 2C. Table S2. Geometrical and materials parameters for the bistable mechanism shown in Fig. 1. Table S3. Geometrical and materials parameters for the optimal bistable mechanism marked in Fig. 2C. Table S4. Mass and size of crawling robot. Table S5. Mass and size of swimming robot.

Supplementary 2 Movie S1. Bistable actuator working under an alternating voltage (3.75 V). Movie S2. Bistable actuator working under different voltages (3.5, 3.75, and 4.0 V). Movie S3. Biased bistable actuator with stop blocks. Movie S4. Bistable crawling robot crawling on the flat ground. Movie S5. Bistable crawling robot crawling on sandpapers. Movie S6. Origami-inspired bistable paddles. Movie S7. Bistable swimming robot.
