# Supplementary material for: A Torsion-Bending Antagonistic Bistable Actuator Enables Untethered Crawling and Swimming of Miniature Robots
Source: Research (Wash D C). 2023 Apr 11;6:0116. doi: 10.34133/research.0116 (PMC10243200; doi:10.34133/research.0116)
Supplement: Supplementary 1 — Note S1. Fabrication and modeling of bistable mechanism. Note S2. Preparation and characterization of IPMC. Note S3. Characterization of bistable actuator. Note S4. Control of bistable actuator. Note S5. Design of origami-inspired paddles. Figure S1. Fabrication process of bistable structure. Figure S2. Torsion and bending of LET beams. Figure S3. Mechanical model of torsion-bending antagonistic bistable mechanism. Figure S4. Stable angles of bistable mechanisms with different geometrical and materials parameters. Figure S5. Experimental setups for testing the stable angle (a) and the moment (b) of the bistable mechanism. Figure S6. Preparation and characterization of IPMC. Figure S7. Characterization of bistable actuator. Figure S8. Control of bistable actuator. Figure S9. Miura-ori structure. Table S1. Theoretical and experimental results of stable angle and maximum moment of bistable designs plotted in Fig. 2C. Table S2. Geometrical and materials parameters for the bistable mechanism shown in Fig. 1. Table S3. Geometrical and materials parameters for the optimal bistable mechanism marked in Fig. 2C. Table S4. Mass and size of crawling robot. Table S5. Mass and size of swimming robot. [file research.0116.f1.docx]

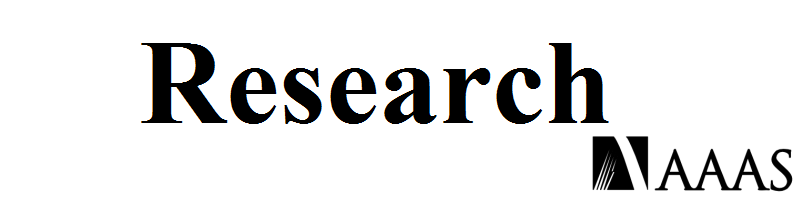


Supplementary Materials for

**A torsion-bending antagonistic bistable actuator enables untethered crawling and swimming of miniature robots**

Nan Hu^1^, Bo Li^1^, Ruiyu Bai^1^, Kai Xie^2^, Guimin Chen^1,*^

^1^ State Key Laboratory for Manufacturing Systems Engineering and Shaanxi Key Lab for Intelligent Robots, School of Mechanical and Engineering, Xi’an Jiaotong University, Xi’an 710049, China.

^2^ School of Aerospace Science and Technology, Xidian University, Xi’an 710126, China.

*Corresponding author. Email: guimin.chen@xjtu.edu.cn

**This file includes:**

Notes S1 to S5

Figures S1 to S9

Tables S1 to S5

Legends for Movies S1 to S7

**Other Supplementary Materials for this manuscript include the following:**

Movies S1 to S7 (.mp4 format)

**Note S1. Fabrication and modeling of bistable mechanism**

**1.1 Fabrication** **of bistable mechanism**

The torsion-bending antagonistic bistable mechanism proposed in this work consists of a pre-stretched lamina emergent torsional (LET) joint and two living hinges. Figure S1 demonstrates the fabrication process of the bistable structure.


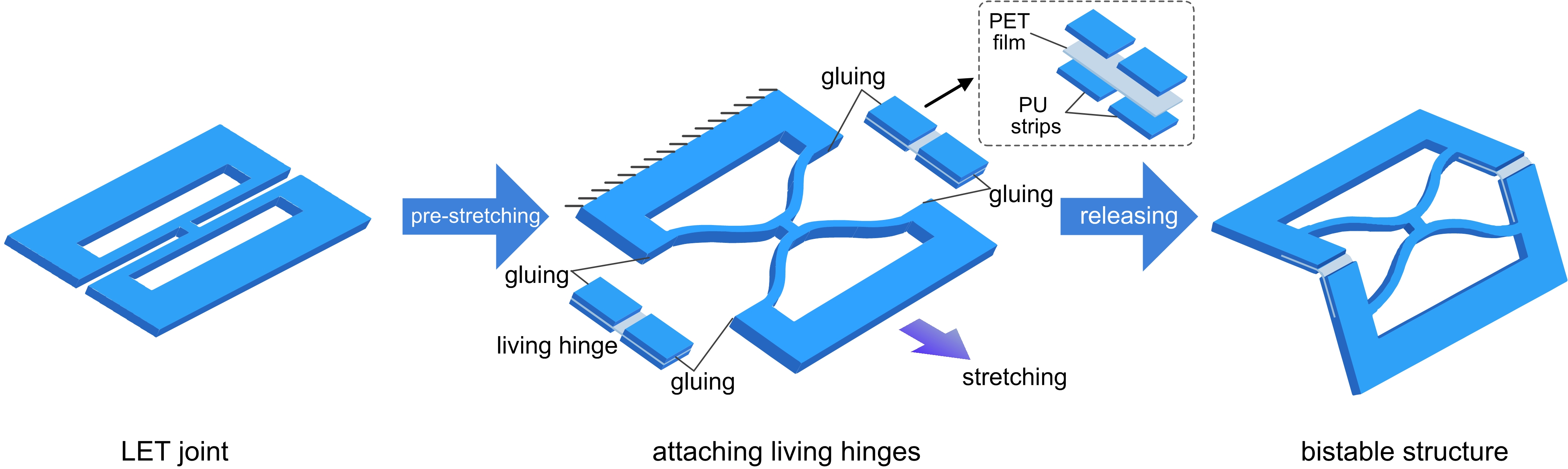


**Figure S1. Fabrication process of bistable structure.**

The living hinge in the bistable structure is consisted of a polyethylene terephthalate (PET) film (thickness: 20 μm) and four polyurethane (PU) strips (thickness: 250 μm). PU strips are glued to the two ends of PET film to constrain the torsional deflection, while only the middle part of PET film keep flexible for bending. We fixed one end of the LET joint and stretched another end in-plane to a desired position, then glued the prepared living hinges in between the rigid frames of the two ends. Once the stretching force is released, the stored strain energy will deflect the bistable structure to one of its two stable states.

**1.2 Modeling of bistable mechanism**

In this work, the chained beam constraint model (CBCM) and the linear torsion model are used to analyze the bending and torsional strain energy of LET beams, respectively.

**1.2.1 Torsional strain energy of LET beam**

When a LET joint subject to a moment $M_{L}$ (Figure S2(b)), the moment-deﬂection behavior of LET joint is characterized by

$M_{L}=k_{eq}\theta$ (S1)

where $k_{_{eq}}$ is an equivalent spring constant based on a combination of the individual pseudo spring constants, and $\theta$ is the rotation angle of LET joint. Taking the bending deflections of rigid segments into account, the equivalent spring constant is [1]

$k_{eq}=\frac{2G_{L}H_{L}^{3}W_{L}^{3}\left(1.17\frac{H_{L}^{2}}{W_{L}^{2}}+2.191\frac{H_{L}}{W_{L}}+1.17\right)}{7L_{L}\left(H_{L}^{2}+W_{L}^{2}\right)\left(\frac{H_{L}^{2}}{W_{L}^{2}}+2.609\frac{H_{L}}{W_{L}}+1\right)}$ (S2)

where $G_{L}$ is the shear modulus of LET beam, and $G_{L}=E_{L}/(2+2\mu_{L})$ ($E_{L}$ is elastic modulus and $\mu_{L}$ is Poisson’s ratio of the LET beam). $L_{L}$, $W_{L}$ and $H_{L}$ are the length, width and thickness of LET beam respectively, as illustrated in Figure S2(a).


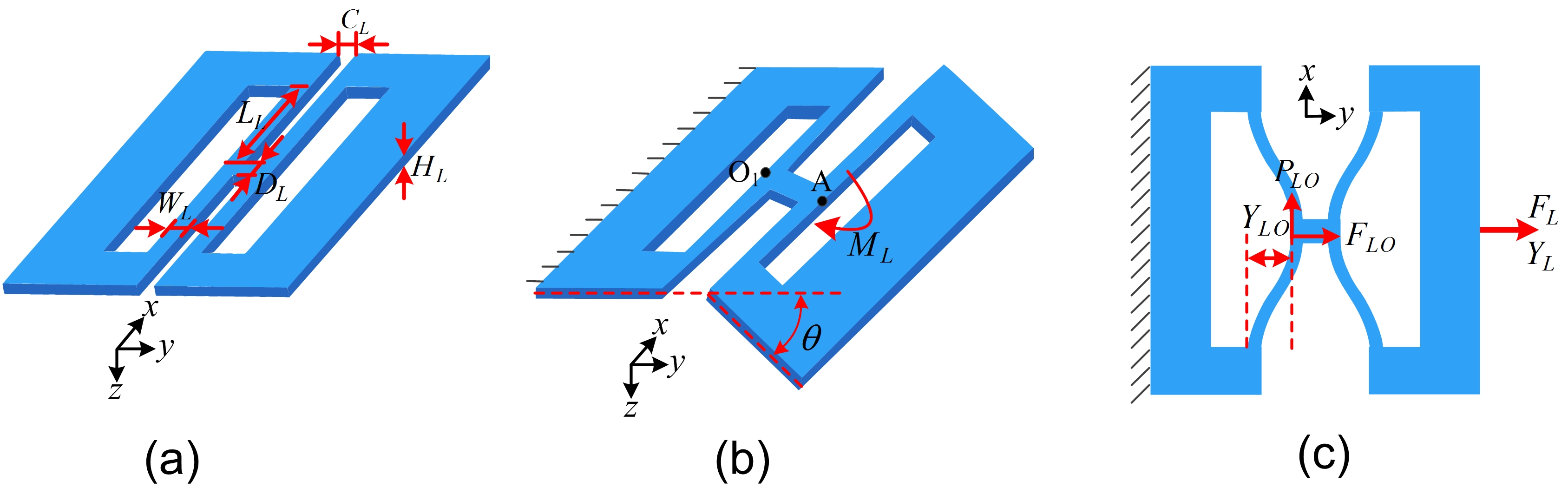


**Figure S2. Torsion and bending of LET beams.** (**a**) Geometric parameters of LET joint. (**b**) Rotation of LET joint. The rotation of LET joint yields the torsion of 4 LET beams. O_1_ is fixed point and A is action point of force or moment. (**c**) Stretching of LET joint. The stretching of LET joint leads to the bending of 4 LET beams.

**1.2.2 Bending strain energy of LET beam**

When the LET joint is subject to a tensile load $F_{L}$ (yielding a deﬂection $Y_{L}$), deflections of the LET beams are identical to each other and they can be treated as ﬁxed-guided segment [2]. The transverse force, axial force and axial deﬂections of one segment are denoted as $F_{LO}$, $P_{LO}$ and $Y_{LO}$ respectively (Figure S2(c)). By using the bi-beam constraint model, the tensile load $F_{L}$ can be expressed as

$F_{L}=\frac{9p_{LO}^{2}+312p_{LO}+720}{2p_{LO}+60}\frac{E_{L}I_{L}}{L_{L}^{3}}Y_{L}$ (S3)

where $I_{L}$ is the area moment of inertia of the LET beam ($I_{L}=W_{L}H_{L}^{3}/12$), $p_{LO}$ is normalized axial force, and

$p_{LO}=\frac{B_{3}-A_{2}}{A_{1}}-\frac{B_{1}}{A_{1}B_{3}}$ (S4)

where

$A_{1}=\frac{4h_{L}^{2}}{225y_{LO}^{2}}+\frac{1}{14000}$, $A_{2}=\frac{16h_{L}^{2}}{45y_{LO}^{2}}-\frac{17}{2100}$, $A_{3}=\frac{16h_{L}^{2}}{3y_{LO}^{2}}-\frac{96}{175}$

$B_{1}=A_{1}A_{3}-A_{2}^{2}$, $B_{2}=1.5A_{1}(A_{2}A_{3}+9.6A_{1})-A_{2}^{3}$, $B_{3}=\left(B_{2}+\sqrt{B_{1}^{3}+B_{2}^{2}}\right)^{1/3}$

$h_{L}=2H_{L}/L_{L}$, $y_{LO}=Y_{L}/L_{L}$

**1.2.3** **Static equilibrium of LET beams**

For the bistable mechanism in Figure S3, point O_1_ is fixed and all loads are applied on point A. A global coordinate frame *xyz* is established on point O_1_ with its *x*-axis along the beam length before deflection, the *y*-axis points from O_1_ to A_O_ (unstable equilibrium state, $\theta=0$) and the *z*-axis perpendicular to the *x* and *y* axes, simultaneously.


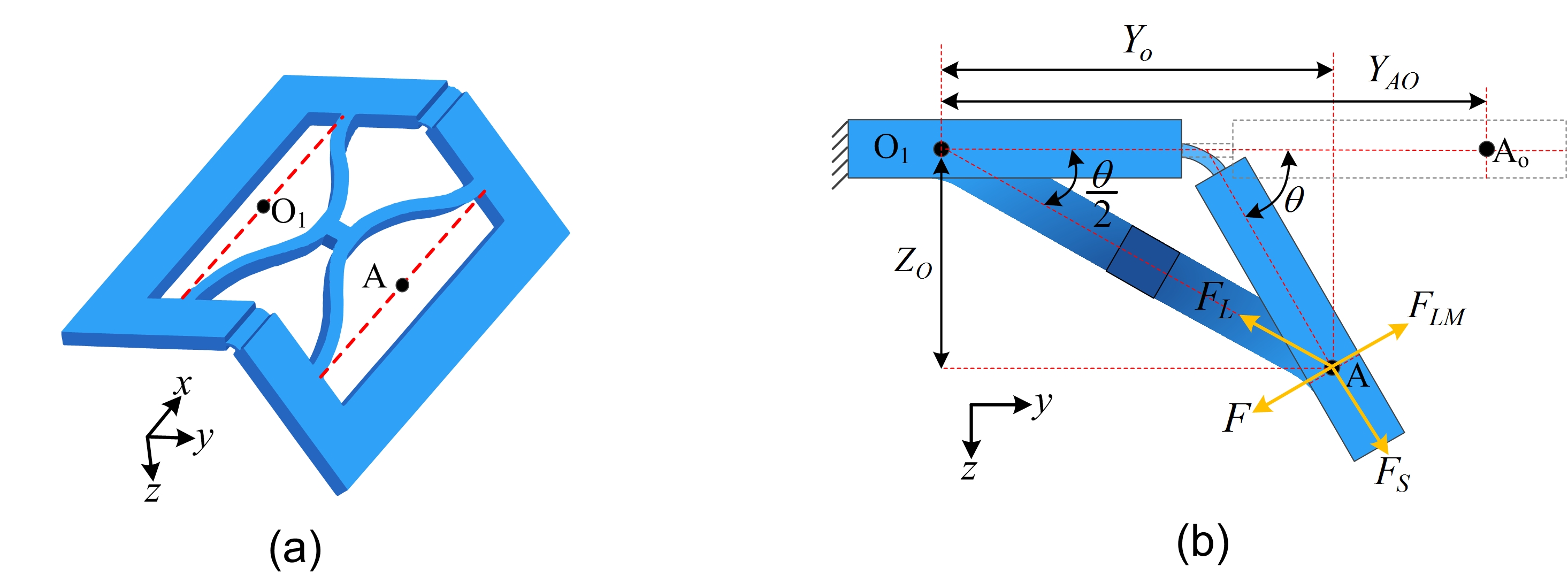


**Figure S3. Mechanical model of torsion-bending antagonistic bistable mechanism.** (**a**) Illustration of bistable mechanism at one stable state. (**b**) Static equilibrium diagram of LET beams. To simplify the mechanical model, the point O_1_ and A (A_o_) are regarded as connecting points of LET beams and frames.

As illustrated in Figure S3(b), the loads on point A from LET beams are $F_{L}$ and $F_{LM}$, the supporting force from living hinge is $F_{S}$, and the force to maintain static equilibrium is $F$ (when the bistable mechanism is on stable state, $F=0$). These forces together cause the point A deflects to position $\left(0,Y_{O},Z_{O}\right)$ with a tilted angle $\theta$. The static equilibrium equations of point A are:

$\left\{\begin{matrix} F_{LM}\cos\left(\frac{\pi}{2}-\theta\right)+F_{S}\cos\left(\theta\right)-F_{L}\cos\left(\frac{\theta}{2}\right)-F\cos\left(\frac{\pi}{2}-\theta\right)=0 \\ F_{LM}\sin\left(\frac{\pi}{2}-\theta\right)-F_{S}\sin\left(\theta\right)+F_{L}\sin\left(\frac{\theta}{2}\right)-F\sin\left(\frac{\pi}{2}-\theta\right)=0 \end{matrix}\right.$ (S5)

According to geometric constraint, the stretch $Y_{L}$ (Figure S2(c)) of LET joint is:

$Y_{L}=\sqrt{Z_{O}^{2}+Y_{O}^{2}}-2W_{L}-C$ (S6)

where $C$ is the length of connecting segment of LET beam (Figure S2(a)). The pre-stretch $Y_{LP}$ of LET joint is:

$Y_{LP}=Y_{AO}-2W_{L}-C$ (S7)

where $Y_{AO}$ is the distance from point $O_{1}$ to $A_{o}$ (shown in Figure S3(b)), and

$Y_{AO}=2Z_{O}\sin\theta$ (S8)

The relation between $Y_{O}$, $Z_{O}$ and $\theta$ is:

$\tan\frac{\theta}{2}=\frac{Z_{O}}{Y_{O}}$ (S9)

The relation between the moment $M_{L}$ (Figure S2(b)) and $F_{LM}$ is:

$M_{L}=F_{LM}Y_{O}/\sin\theta$ (S10)

and the relation between the moment $T$ to maintain static equilibrium (Figure 2(c)) and $F$ is:

$T=FY_{O}/\sin\theta$ (S11)

Equations (S1), (S3) and (S5) to (S11) constitute the static equilibrium equations (totally 10 equations) for the bistable mechanism. Among 12 parameters $F_{L}$, $F_{LM}$, $F_{S}$, $F$, $M_{L}$, $T$, $Y_{O}$, $Z_{O}$, $\theta$, $Y_{LP}$, $Y_{AO}$ and $Y_{L}$, given $\theta$ and $Y_{LP}$, the other 10 can be obtained by solving the static equilibrium equations numerically.

**1.2.4 Bistability analysis of bistable mechanism**

The stable angles of bistable mechanisms fabricated with different materials (PU: polyurethane, PP: polypropylene, PET: polyethylene terephthalate, PC: polycarbonate) are listed in Figure S4(a). It shows that the value of stable angle increases with the increase of elastic modulus. However, the feasible range of the bending angle of ionic polymer metal composite (IPMC) used in this work is no more than 18.5°, and it fails to meet the requirement of bistable actuation if the bistable mechanism is fabricated with material of PP, PET or PC. In addition, PU features high Poisson's ratio and toughness, which offers an advantage that the LET beams made of PU are easy to be twisted. Therefore, the PU material is selected to fabricate the LET joint.


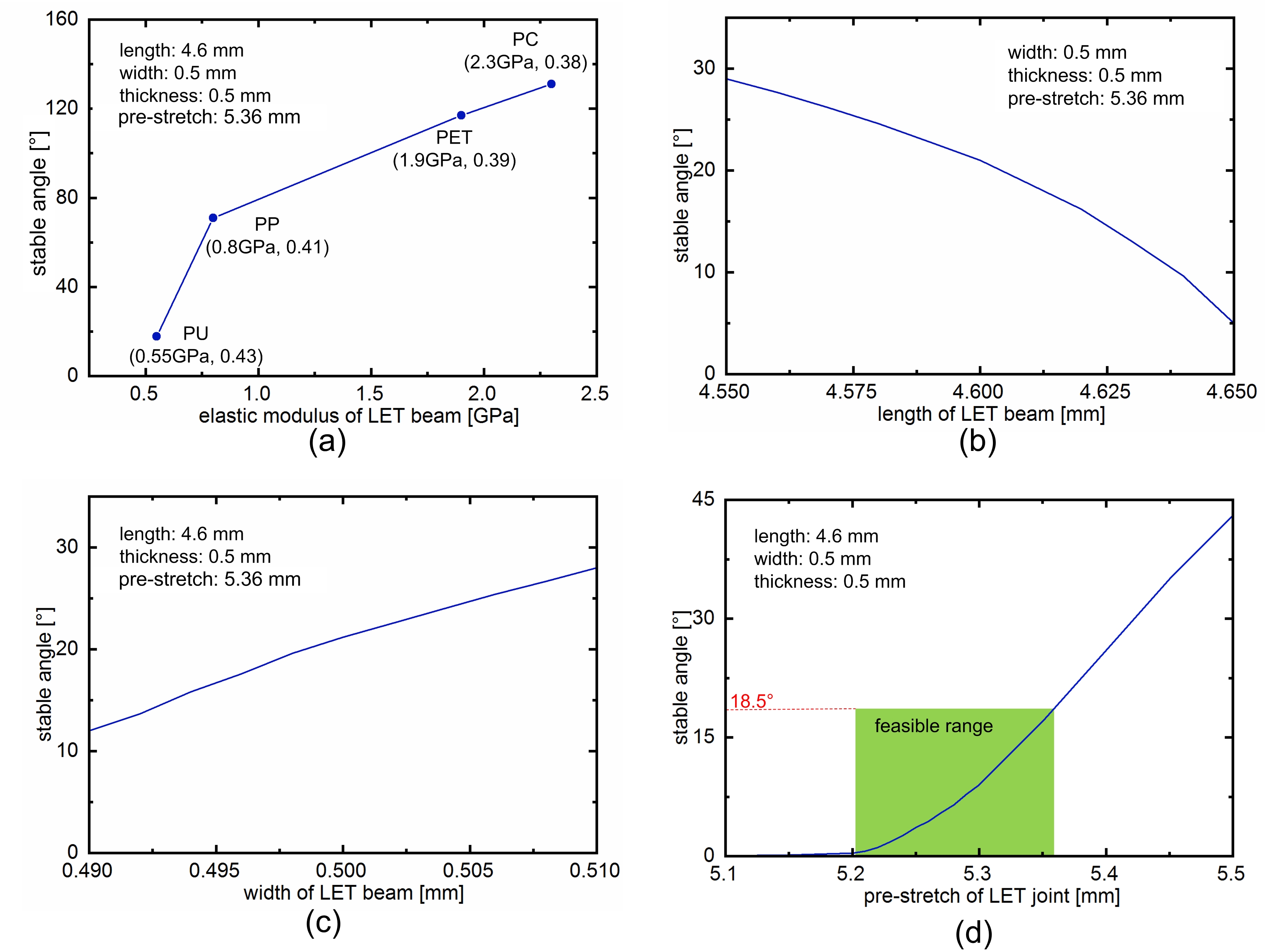


**Figure S4. Stable angles of bistable mechanisms with different geometrical and materials parameters.** (**a**) Stable angles of bistable mechanisms with different materials. The elastic modules and Poisson's ratio are labeled below the name of the material. (**b**), (**c**) and (**d**) are the stable angles of bistable mechanisms with different lengths and widths of LET beams and different pre-stretch levels of LET joints respectively.

The stable angles of the bistable mechanisms with different lengths and widths of LET beams and different pre-stretch levels of LET joints are shown in Figures S4(b), (c) and (d). It can be seen that the stable angles are enlarged with the increase of the width of LET beam and the pre-stretch of LET joint, while reduced with the increase of the length of LET beam.

As shown in Figure S5, a laser displacement sensor (IL-065, Keyence) and a force sensor (GSO-10, Transducer Techniques) are utilized to measure the stable angle $(\theta=2\arctan(x/l))$ and maximum moment ($T=fl$) of the bistable mechanism, respectively, and the distance from the test point to the fixed end is 10 mm (*l*), the theoretical and the experimental results are listed in Table S1.


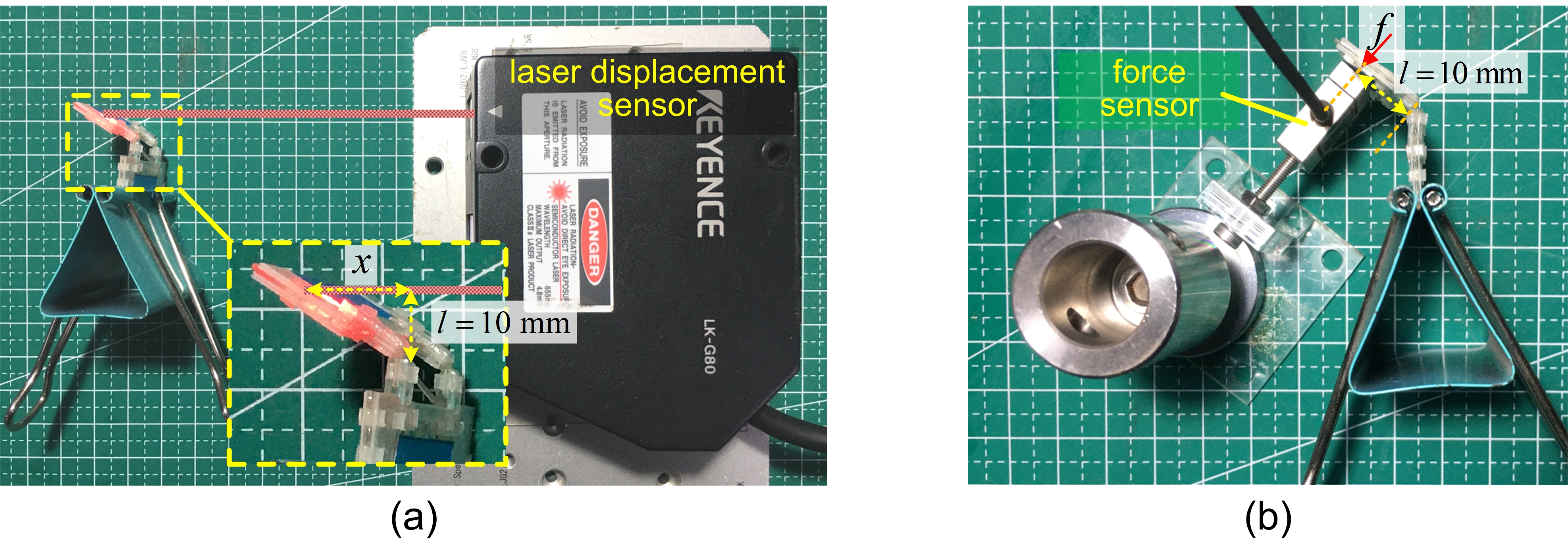


**Figure S5. Experimental setups for testing the stable angle (a) and the moment (b) of the bistable mechanism.**

**Note S2. Preparation and characterization of IPMC**

IPMC is one of the ionic electroactive materials in a strip shape which can bend in the thickness direction under a low voltage (usually 3 V to 5 V), it consists of an ion exchange layer and two electrode layers (Figure S6(a)). The hydration cations and anions in the ion exchange membrane of IPMC are uniformly distributed when power is off. When an external voltage is applied across its two electrodes, the anions (small size) migrate towards the anode side and the hydration cations (large size) migrate towards the cathode side, which leads to a remarkably greater expansion of the cathode than that of the anode side, resulting in the bending deformation of IPMC.


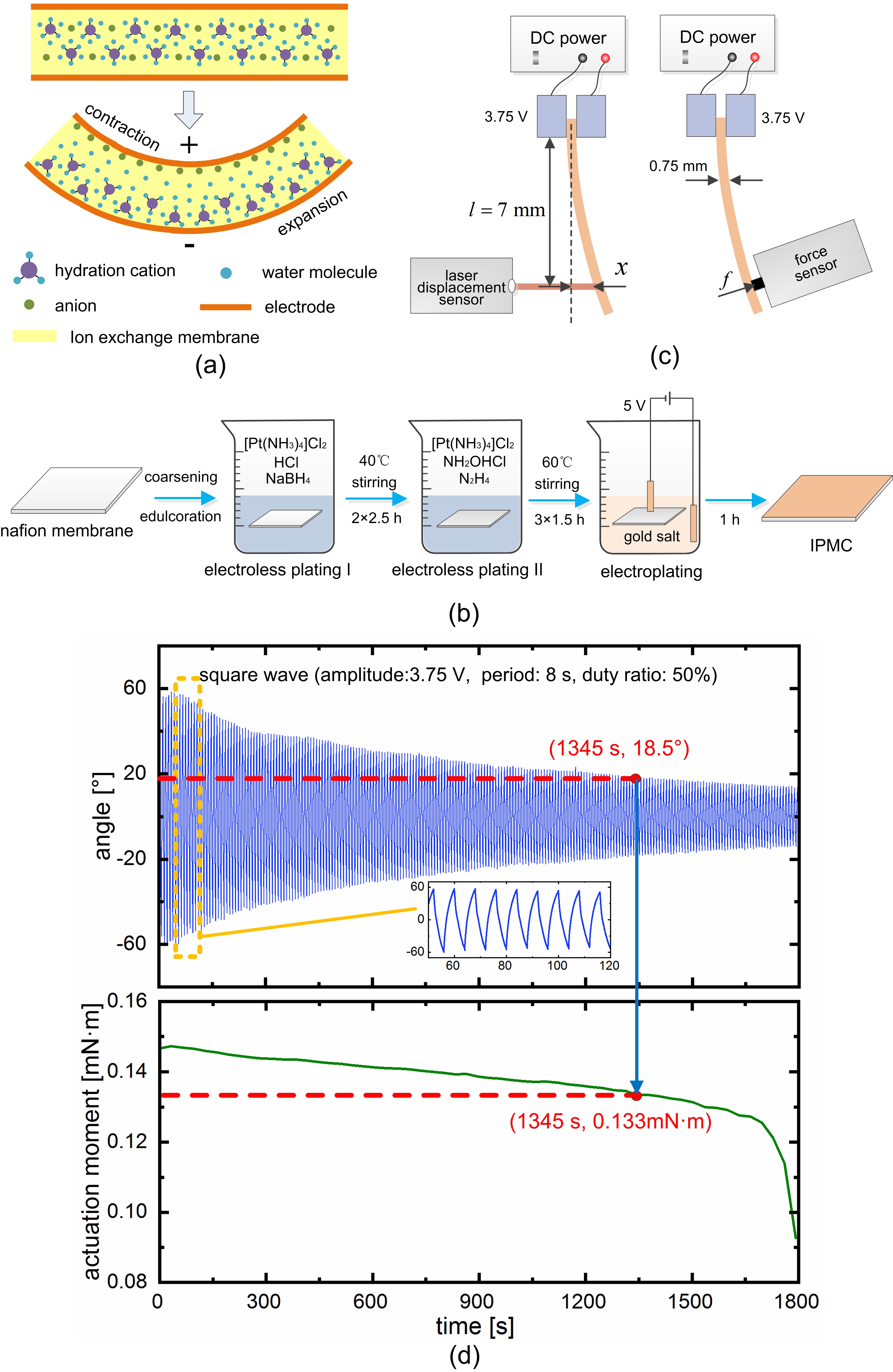


**Figure S6. Preparation and characterization of IPMC.** (**a**) Actuation principle of IPMC. (**b**) Preparation process of IPMC (ion exchange membrane: Nafion, electrode: palladium). (**c**) Experimental setup for testing the deflection and the actuation moment of IPMC. (**d**) Characterization of IPMC under a voltage of 3.75 V.

Preparation steps of IPMC are as follow (Figure S6(b)):

(1) Surface coarsening of Nafion membrane (50 mm × 50 mm × 0.75 mm). This step increases membrane surface area to enhance the adhesion between the electrodes and the membrane.

(2) Electroless plating I. An initial palladium electrode is fabricated on the surface of the membrane by electroless plating. This step usually are repeated 2~3 times.

(3) Electroless plating II. This step fabricates more palladium to enhance its electrical conductivity as electrode.

(4) Electroplating. In this step, the gold ions are plated on the outermost layer to further improve the conductivity and finalize a protective layer for encapsulating.

The experimental setup for testing the performance of IPMC are illustrated in Figure S6(c), a laser displacement sensor (IL-065, Keyence) and a force sensor (GSO-10, Transducer Techniques) are utilized to measure the deflection angle $(\theta=2\arctan(x/l))$ and actuation moment ($T=fl$) of IPMC respectively, and the distance from the test point to the fixed end is 7 mm.

Figure S6(d) plots the bending angle and the actuation moment of an IPMC strip by applying a voltage of 3.75 V. The bending angle should be greater than 18.5° for the IPMC to switch the bistable mechanism between two stable states. From Figure S6(d), it is observed that the bending angle gradually reduces to 18.5° at 1345 s, and the corresponding actuation moment is 0.133 mN•m. The total actuation moment provided by the two IPMC strips (which are symmetrically placed in the bistable structure) is beyond the required moment to switch the bistable mechanism (0.26 mN•m). Therefore, the lifetime of the IPMC can be approximately determined as 22 min. The driving voltage and average current are 3.75 V and 100 mA, respectively, and the estimated power consumption of IPMC (voltage multiplied by current) is 375 mW.

**Note S3. Characterization of bistable actuator**

A laser displacement sensor (IL-065, Keyence) and a force sensor (GSO-10, Transducer Techniques) are utilized to measure the deflection angle $(\theta=2\arctan(x/l))$ and actuation moment ($T=fl$), respectively, and the distance from the test point to the fixed end is 17.5 mm (*l*). The experimental setups are illustrated in Figures S7(a) and (b). The experimental results of actuation moment are shown in Figures S7(d) and (e). Increasing both the voltage and the normalized pre-stretch leads to increase of the actuation moment of the bistable actuator.

The rotation angle and angular velocity of bistable actuator in the lifetime test are plotted in Figure S7(c), which shows that the lifetime of the actuator under voltages of 3.5 V, 3.75 V and 4.0 V are 17 min, 15 min and 7 min, and the average actuation period during the corresponding lifetime are 40 s, 29 s and 47 s, respectively.


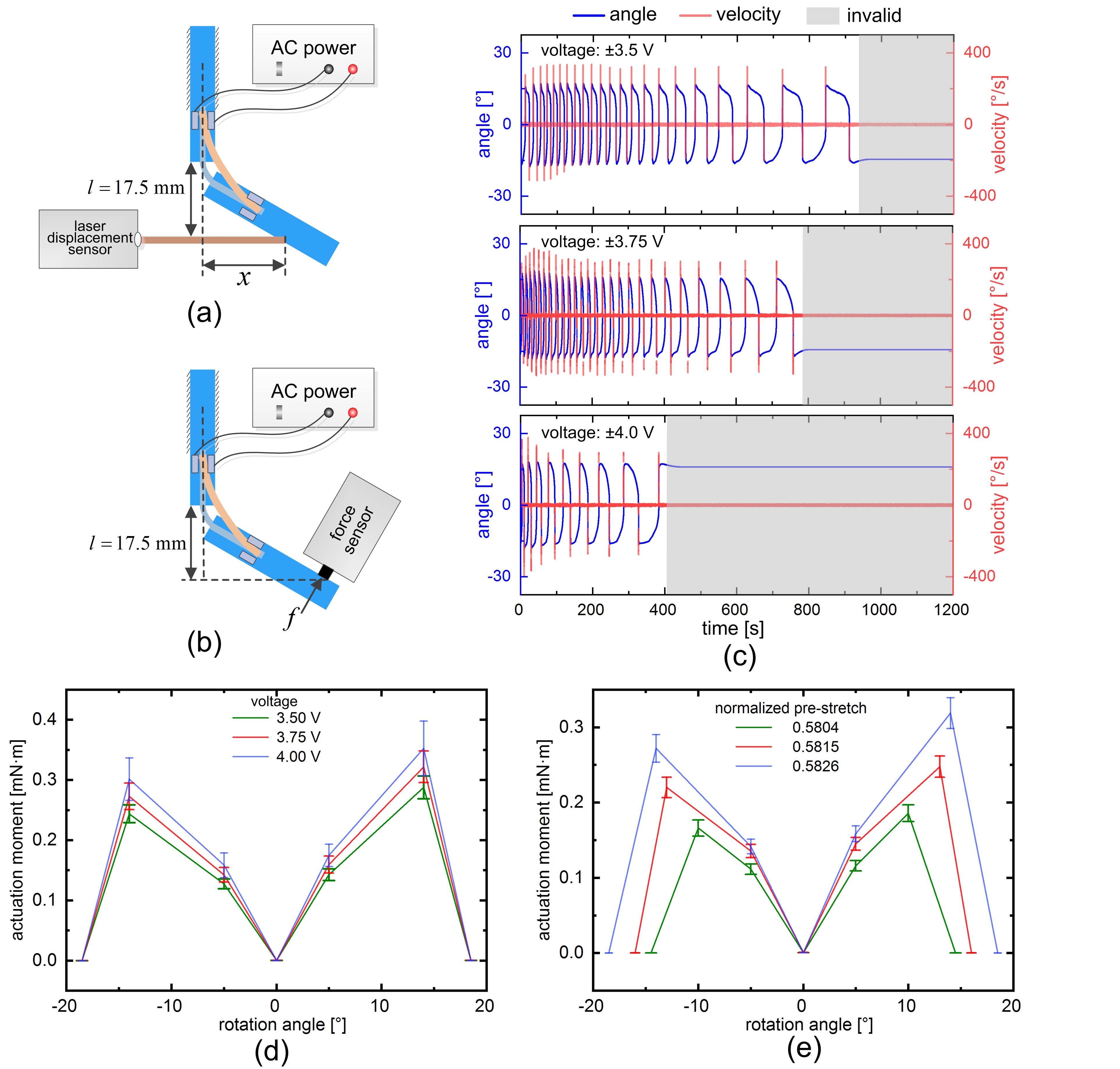


**Figure S7. Characterization of bistable actuator.** (**a**) and (**b**) Experimental setup for testing the rotation angle and the actuation moment of bistable actuator. (**c**) Rotation angle and angular velocity of bistable actuator with voltages of different amplitude (3.5 V, 3.75 V and 4 V). (**d**) Actuation moment of bistable actuator with voltages of different amplitude (3.5 V, 3.75 V and 4 V). (**e**) Actuation moment of bistable actuator with different normalized pre-stretch (0.5804, 0.5815 and 0.5826).

**Note S4. Control of bistable actuator**

Figure S8(a) illustrates the control circuit employed to determine the actuation period of IPMC, which consists of a tiny Microcontroller (MCU, ST-stm32F401), a Chopper Driver (Allergo-A3908) and a Current Shunt (0.1 Ω). The MCU periodically detects the current in IPMC at an interval of 0.1 s and toggles the Chopper Driver when the deviation between 5 consecutive sampled values is less than a threshold of 0.02 mA (which was determined experimentally, Figure S8(b)) for the bistable actuator to rotate in the reverse direction.


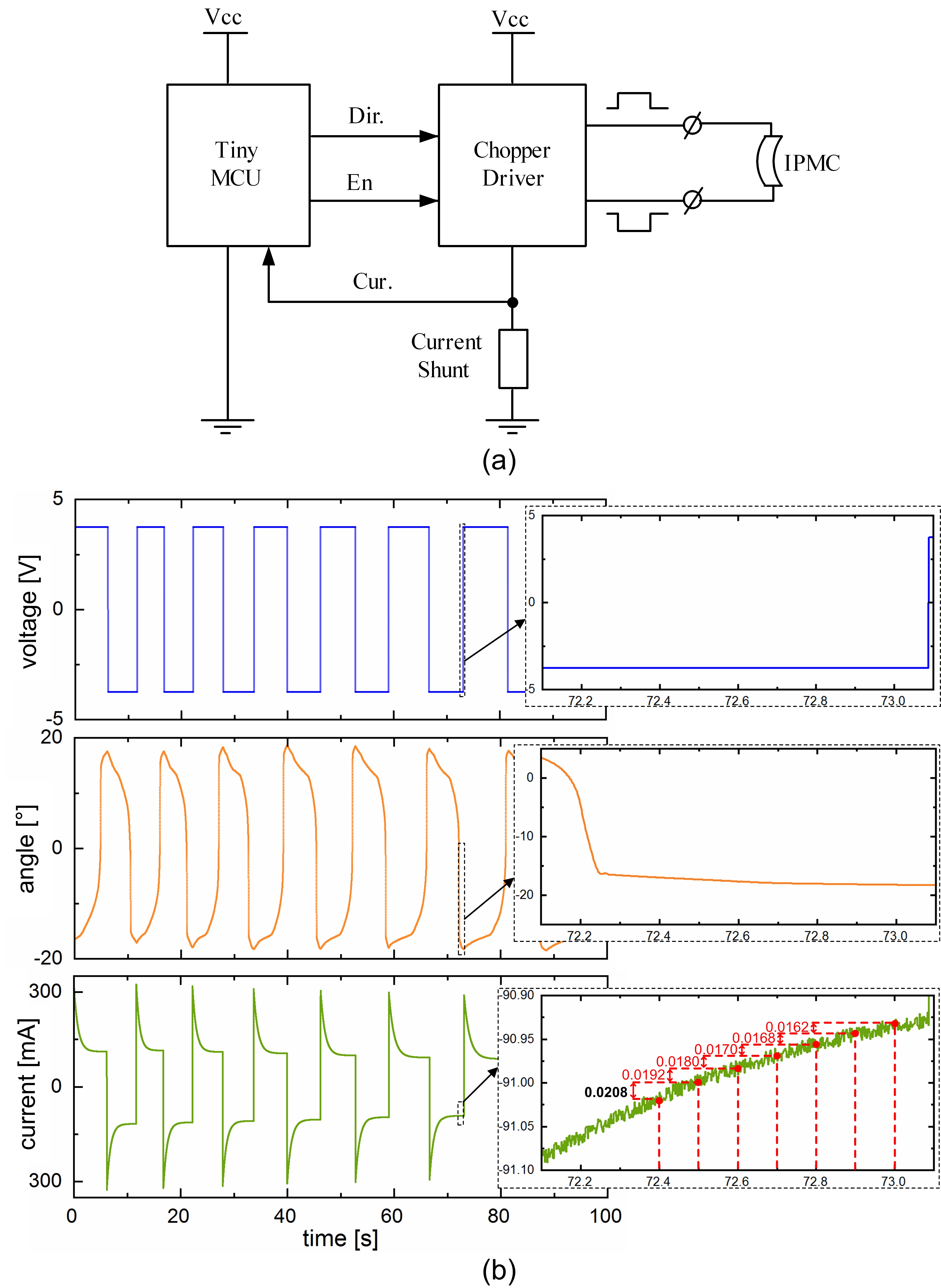


**Figure S8. Control of bistable actuator.** (**a**) Diagram of control circuit. (**b**) Measured current in IPMC implanted in bistable actuator.

**Note S5. Design of origami-inspired paddles**

As one of the classic origami-inspired structures, Miura-ori can be modeled as a spatial linkage mechanism. As shown in Figure S9(a), the simplest Miura-ori with 1 vertex and 4 creases (3 valley creases and 1 mountain crease) enables to amplify the stroke of the bistable actuator. According to the spatial geometric relation [3], the rotation angles of the creases in a Miura-ori structure are:

(S12)


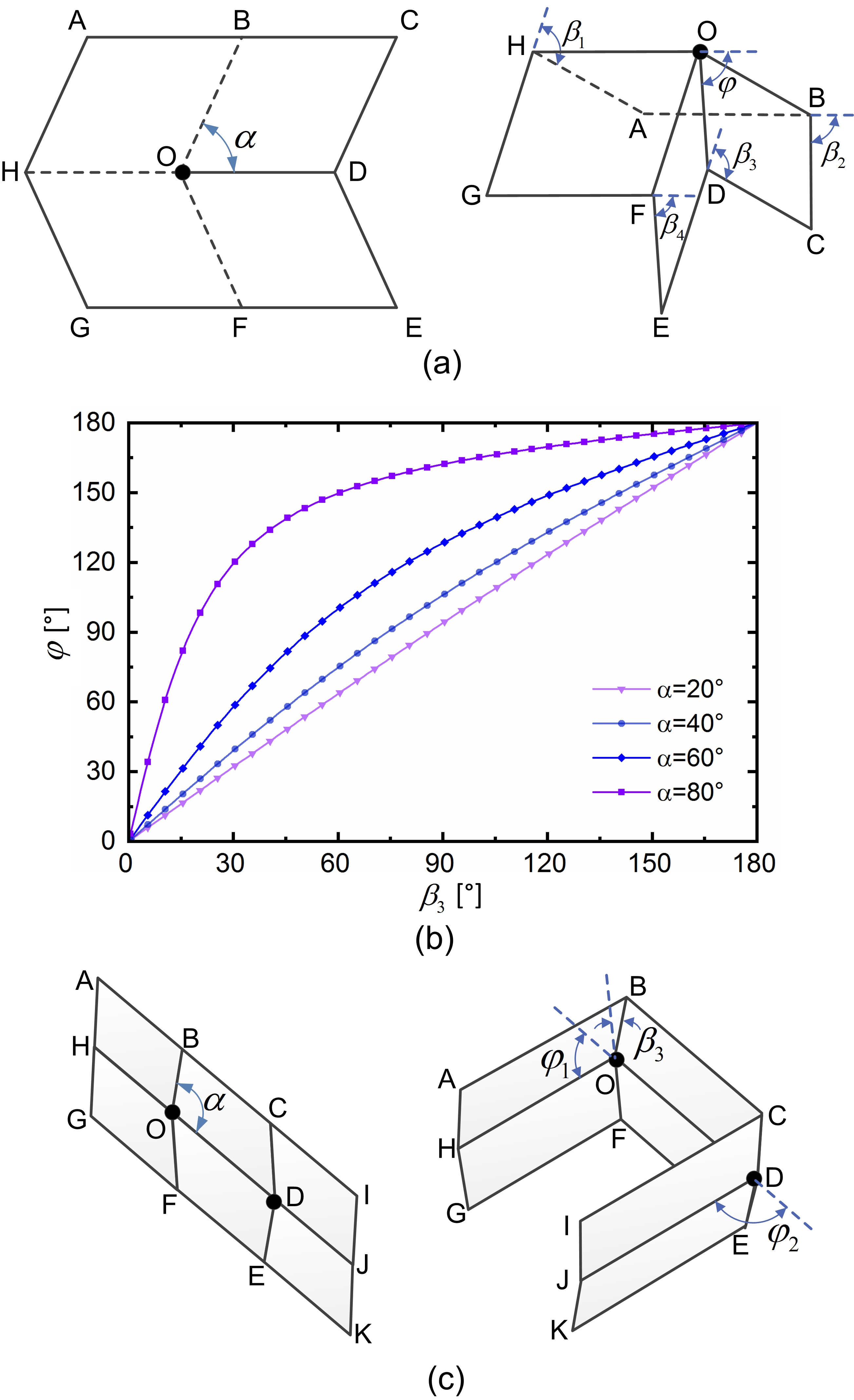


**Figure S9. Miura-ori structure.** (**a**) Four-crease Miura-ori (one vertex, three valley creases and one mountain crease). (**b**) changes with $\beta_{3}$ when $\alpha$ are 20°, 40°, 60° and 80°. (**C**) Seven-crease Miura-ori (two vertexes, six valley crease and one common mountain crease). $\beta_{3}$ is the input angle (actuated by bistable actuator) and is the output angle.

Figure S9(b) plots the changes of with $\beta_{3}$ when $\alpha$ are 20°, 40°, 60° and 80°, it can be seen that $\beta_{3}$ is always greater than . Therefore, this origami-inspired structure can amplify the rotational motion, and the magnification increases with the increasing of $\alpha$.

Figure S9(c) is a Miura-ori with two vertexes and seven creases (six valley creases and one mountain crease). This structure has two symmetrical and equal rotation angles ( and ) in its left and right sides. $\beta_{3}$ is the input angle actuated by the bistable actuator, then and are output angles. In this work, angle $\alpha$ is selected as 80°. This structure can amplify the stroke of the bistable actuator from 20.5° ($\beta_{3}$) to 94° ( and ).

**Table S1. Theoretical and experimental results of stable angle and maximum moment of bistable designs plotted in Figure 2(c)**

| **Aspect ratio** | **Normalized pre-stretch** | **Stable angle** | | | **Maximum moment** | | |
| --- | --- | --- | --- | --- | --- | --- | --- |
|  |  | theoretical  *θ*_t_ [°] | experimental  *θ*_e_ [°] | relative error $\left\vert\theta_{\text{e}}-\theta_{\text{t}}\right\vert/\theta_{\text{t}}$ | theoretical  *T*_tmax_ [mN·m] | experimental  *T*_emax_ [mN·m] | relative error $\left\vert T_{\text{emax}}-T_{\text{tmax}}\right\vert/T_{\text{tmax}}$ |
| 0.98 | 0.595 | 33.0 | 32.1 | 2.6% | 0.503 | 0.497 | 1.1% |
| 0.98 | 0.590 | 27.5 | 28.2 | 2.4% | 0.281 | 0.271 | 3.7% |
| 0.98 | 0.585 | 19.5 | 20.2 | 3.5% | 0.109 | 0.105 | 3.5% |
| 0.98 | 0.580 | 6.0 | 5.8 | 2.8% | 0.010 | 0.010 | 1.2% |
| 0.98 | 0.575 | / | / | / | / | / | / |
| 0.99 | 0.595 | 35.5 | 35.1 | 1.1% | 0.622 | 0.649 | 4.4% |
| 0.99 | 0.59 | 30.0 | 29.1 | 3.0% | 0.380 | 0.384 | 1.2% |
| 0.99 | 0.585 | 23.5 | 23.9 | 1.7% | 0.183 | 0.193 | 5.4% |
| 0.99 | 0.58 | 13.8 | 13.7 | 0.3% | 0.044 | 0.045 | 0.4% |
| 0.99 | 0.575 | / | / | / | / | / | / |
| 1.00 | 0.595 | 37.5 | 37.6 | 0.3% | 0.747 | 0.753 | 0.8% |
| 1.00 | 0.590 | 32.5 | 31.7 | 2.4% | 0.487 | 0.472 | 3.0% |
| 1.00 | 0.585 | 26.5 | 27.2 | 2.8% | 0.268 | 0.261 | 2.5% |
| 1.00 | 0.580 | 19.0 | 18.1 | ***5.0%*** | 0.100 | 0.102 | 1.9% |
| 1.00 | 0.575 | / | / | / | / | / | / |
| 1.01 | 0.595 | 39.3 | 38.8 | 1.2% | 0.880 | 0.834 | 5.3% |
| 1.01 | 0.590 | 35.0 | 35.1 | 0.2% | 0.602 | 0.627 | 4.1% |
| 1.01 | 0.585 | 29.8 | 28.8 | 3.1% | 0.364 | 0.358 | 1.5% |
| 1.01 | 0.580 | 22.8 | 23.4 | 2.9% | 0.171 | 0.154 | ***9.0%*** |
| 1.01 | 0.575 | 10.0 | 10.0 | 0.4% | 0.014 | 0.013 | 3.4% |
| 1.02 | 0.595 | 40.0 | 38.5 | 3.8% | 1.019 | 1.077 | 5.7% |
| 1.02 | 0.590 | 37.0 | 37.4 | 1.2% | 0.724 | 0.748 | 3.2% |
| 1.02 | 0.585 | 32.5 | 31.8 | 2.2% | 0.467 | 0.487 | 4.2% |
| 1.02 | 0.580 | 26.5 | 26.0 | 2.1% | 0.253 | 0.249 | 1.7% |
| 1.02 | 0.575 | 16.0 | 16.1 | 0.7% | 0.057 | 0.054 | 5.2% |

**Table S2. Geometrical and materials parameters for the bistable mechanism shown in Figure 1**

| **Parameter** | | **Symbol** | **Value** | **Unit** |
| --- | --- | --- | --- | --- |
| **LET**  **beam** | length | $L_{L}$ | 5 | mm |
|  | width | $W_{L}$ | 1.5 |  |
|  | thickness | $H_{L}$ | 1.5 |  |
|  | pre-stretch | $Y_{L}$ | 2.7 |  |
|  | Young’s modulus | $E_{L}$ | 800 | MPa |
|  | Poisson's ratio | $\mu_{L}$ | 0.3 |  |

**Table S3. Geometrical and materials parameters for the optimal bistable mechanism marked in Figure 2(c)**

| **Parameter** | | **Symbol** | **Value** | **Unit** |
| --- | --- | --- | --- | --- |
| **LET**  **Beam** | length | $L_{L}$ | 4.6 | mm |
|  | width | $W_{L}$ | 0.5 |  |
|  | thickness | $H_{L}$ | 0.5 |  |
|  | pre-stretch | $Y_{L}$ | 5.36/2 |  |
|  | Young’s modulus  (PU film) | $E_{L}$ | 550 | MPa |
|  | Poisson's ratio  (PU film) | $\mu_{L}$ | 0.42 |  |
| **IPMC**  **strip** | length | $L_{I}$ | 7.2 | mm |
|  | width | $W_{I}$ | 2 |  |
|  | thickness | $H_{I}$ | 0.75 |  |
|  | Young’s modulus | $E_{I}$ | 1100 | MPa |
|  | Poisson's ratio | $\mu_{I}$ | 0.36 |  |

**Table S4. Mass and size of crawling robot**

| Total | 2.69 g | | | | |
| --- | --- | --- | --- | --- | --- |
| Bistable actuator | 0.20 g | | Lithium battery | | 0.75 g |
| Control circuit | 0.68 g | | Body | | 0.58 g |
| Foot (3) | 0.48 g | |  | |  |
|  | | **Body** | material | PET  (thickness 0.5 mm) | |
|  |  | 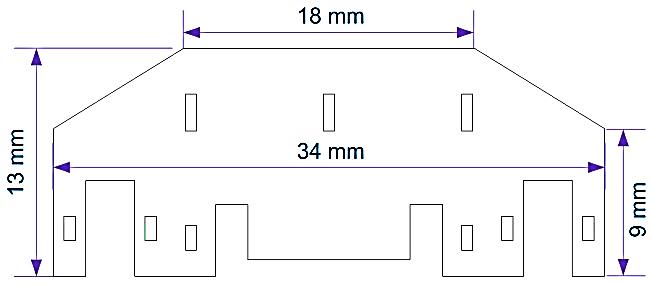 | | | |
|  |  | **Foot** | material | acrylic plate  (thickness 0.8 mm) | |
|  |  | 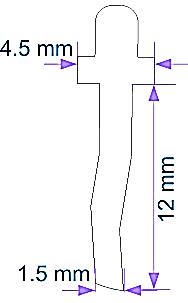 | | | |

**Table S5. Mass and size of swimming robot**

| **Swimming robot** | | | | | |
| --- | --- | --- | --- | --- | --- |
| Total | 7.22 g | | | | |
| Bistable actuator | 0.20 g | | Lithium battery | | 1.20 g |
| Control circuit | 0.68 g | | Body | | 3.70 g |
| Paddles | 1.44 g | |  | | |
| 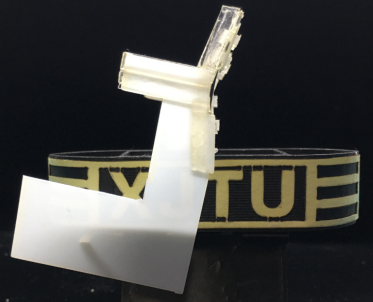 | | **Body** | carbon fibre (3D printing) | | |
|  |  | 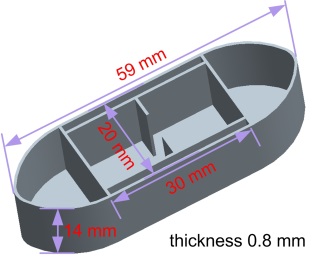 | | | |
|  |  | **Paddles** | | PET (thickness 0.3 mm) | |
|  |  | 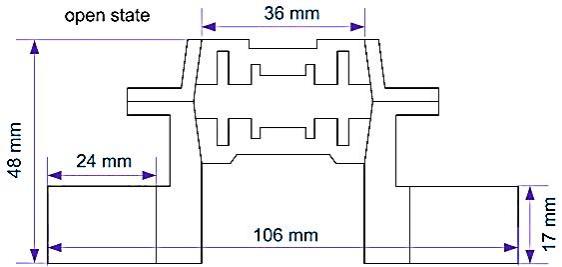 | | | |

**Legends for Movies S1 to S7**

**Movie S1. Bistable actuator working under an alternating voltage (3.75 V).** The bistable actuator has an instantaneous angular velocity of 300 °/s and remains the stable state even the voltage is off.

**Movie S2. Bistable actuator working under different voltages (3.5 V, 3.75 V, and 4.0 V).** The average switching frequency of the bistable actuator (in 150 s) under a voltage of 3.75 V is higher than that of 3.5 V and 4 V, and the voltage amplitude for bistable actuator in this study is selected as 3.75 V.

**Movie S3. Biased bistable actuator with stop blocks.** The rotation angle of the bistable actuator with stop blocks is between −18.5° and 2° (Movie S3), and the switching frequency is twice of that without stop blocks.

**Movie S4. Bistable crawling robot crawling on the flat ground.** The robot can crawl on a flat ground with a maximum instantaneous velocity of 40 mm/s due to the strain energy releasing of the LET beams, and no backwards movement is observed.

**Movie S5. Bistable crawling robot crawling on sandpapers.** To demonstrate the ability to crawl on different grounds, the crawling robot was tested on sandpapers with different grit number of 240, 300 and 400.

**Movie S6. Origami-inspired bistable paddles.** The origami-inspired paddles are actuated by the biased bistable actuator. The paddle angles (*φ*_1_ and *φ*_2_) are between −2° and 92° and are almost synchronous.

**Movie S7. Bistable swimming robot.** The robot can swim breaststroke with a maximum instantaneous velocity of 50 mm/s due to the high propelling force produced by the strain energy releasing of bistable actuator, while the robot without bistable actuator cannot swim forward due to low propelling force. To demonstrate the propelling capacity of the bistable actuator, the robot is loaded with 6 g water.

**Reference**

[1] J. O. Jacobsen, G. Chen, L. L. Howell, S. P. Magleby, Lamina Emergent Torsional (LET) Joint. *Mechanism and Machine Theory* **44**, 2098-2109 (2009).

[2] F. Ma, G. Chen, Modeling Large Planar Deflections of Flexible Beams in Compliant Mechanisms Using Chained Beam-Constraint-Model. *Journal of Mechanisms and Robotics* **8**, 021018 (2016).

[3] J. Butler et al., A Model for Multi-Input Mechanical Advantage in Origami-Based Mechanisms. *Journal of Mechanisms and Robotics* **10**, 061007 (2018).
